# A Coupled Discrete/Continuum Model for Describing Cancer-Therapeutic Transport in the Lung

**DOI:** 10.1371/journal.pone.0031966

**Published:** 2012-03-12

**Authors:** Karin Erbertseder, Johannes Reichold, Bernd Flemisch, Patrick Jenny, Rainer Helmig

**Affiliations:** 1 Department of Hydromechanics and Modeling of Hydrosystems, Institute for Modelling Hydraulic and Environmental Systems, University of Stuttgart, Stuttgart, Germany; 2 Department of Mechanical and Process Engineering, Institute of Fluid Dynamics, ETH Zurich, Zurich, Switzerland; Pennsylvania State University, United States of America

## Abstract

We propose a computational simulation framework for describing cancer-therapeutic transport in the lung. A discrete vascular graph model (VGM) is coupled to a double-continuum model (DCM) to determine the amount of administered therapeutic agent that will reach the cancer cells. An alveolar cell carcinoma is considered. The processes in the bigger blood vessels (arteries, arterioles, venules and veins) are described by the VGM. The processes in the alveolar capillaries and the surrounding tissue are represented by a continuum approach for porous media. The system of equations of the coupled discrete/continuum model contains terms that account for degradation processes of the therapeutic agent, the reduction of the number of drug molecules by the lymphatic system and the interaction of the drug with the tissue cells. The functionality of the coupled discrete/continuum model is demonstrated in example simulations using simplified pulmonary vascular networks, which are designed to show-off the capabilities of the model rather than being physiologically accurate.

## Introduction

According to the World Health Organization, lung cancer kills more people than any other type of cancer and is responsible for 1.4 million deaths worldwide yearly [1]. Often, drug treatments employ a trial and error procedure to determine the most effective dosage. A predictive mathematical model suitable to guide cancer-therapeutic strategies is still lacking. There exist plenty of publications about the modeling of fluid flow and delivery of macromolecules in solid tumors, for example: [2], [3], [4], [5], [6] and [7]. Further, there are several publications about blood flow simulations in vascular networks, for example: [8], [9], [10], [11] and [12]. While the application of these models is restricted to tumor tissue or to vascular networks, the modeling concept presented here is designed for the simulation of the fluid and drug transport in the entire organ affected by the cancer: the macrocirculation, the microcirculation, the tissue and the tumor. A mathematical and a numerical model are developed that describe the distribution of a targeted protein therapeutic within the human lung for cancer therapy. The developed model concept is based on these former publications about the flow and transport processes in the macrocirculation, in the microcirculation and in tumors. However, the coupling of a model for the macrocirculation to a second model for the microcirculation and the surrounding tissue and the representation of a whole organ affected by a tumor are new.

To model the delivery of the therapeutic agent to the tumor cells, the transport of the dissolved drug molecules within the blood vessels, the flow across the vasculature walls into the surrounding tissue, and the transport through the interstitial space towards the tumor have to be described. If the tumor exceeds a diameter of about three millimeters, tumor induced angiogenesis will occur [13]. In this case, a direct transport of the therapeutic agent via the blood vessels to the targeted cells is possible. The model has to account for all aforementioned modes of transport. The development of a mathematical and a numerical model that are suitable to guide lung cancer therapeutic strategies is an ambitious aim. This work does not claim to fully achieve this ultimate goal. However, it is a first step towards it. This paper focuses on the model development taking into account a number of simplifying assumptions.

**Figure 1:**
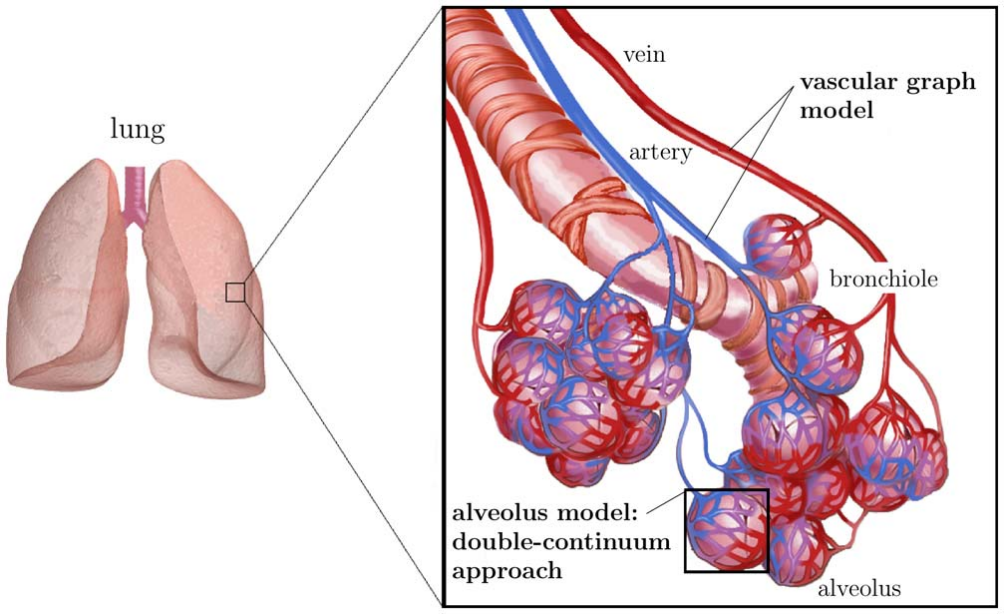
General model concept. The vascular graph model describes the processes occurring in the arteries, arterioles, venules and veins. The alveolus model, a double-continuum approach, represents the processes occurring in the capillary bed and the surrounding tissue (right image according to Terese Winslow).

Figure 1 depicts the general concept of the model. It includes the transport of the injected therapeutic agent through the pulmonary circulation, the transition of the dissolved drug molecules from the blood vessels into the tissue and the processes occurring within the pulmonary tissue. The advection and reaction of the blood-dissolved drug within the non-capillary part of the vasculature is simulated using the previously presented vascular graph model (VGM, see Section 1.1 and [9]). The abundance of pulmonary capillaries (about 1800 capillary segments per alveolus [14]) prevents the application of this discrete approach to the capillary bed due to the high computational cost incurred. Therefore, the flow, transport and reaction processes within the capillary bed and the surrounding tissue are described by the alveolus model instead, which is a double-continuum approach (see Section 1.2). This approach is based on two separated continua: the pulmonary tissue, and the pulmonary capillaries that are coupled by transfer functions (see Section 1.2.4). Thus, so-called upscaled nodes are inserted into the computational lattice of the VGM, which represent the capillary bed described by the alveolus model. In this way, the VGM blood flow simulations are corrected for the loss of therapeutic agent by the transfer of the dissolved drug molecules through the capillary walls into the tissue. The coupling of the alveolus model and the vascular graph model is described in more detail in Section 1.3. An alveolar cell carcinoma (cancer cells located in the alveolar tissue) is modeled by introducing two kinds of upscaled nodes, representing healthy and tumor tissue respectively. The concentration distribution of a therapeutic agent administered via a bolus injection is determined within the blood vessel network and the surrounding tissue. Due to the different physiological properties in a tumor, the drug concentration in the cancer region differs from the one in the healthy pulmonary tissue. The simulation results, which demonstrate the functionality of the coupled discrete/continuum model, are presented in Section 2.4.

## Methods

### 1.1 Vascular Graph Model (VGM)

**Figure 2:**
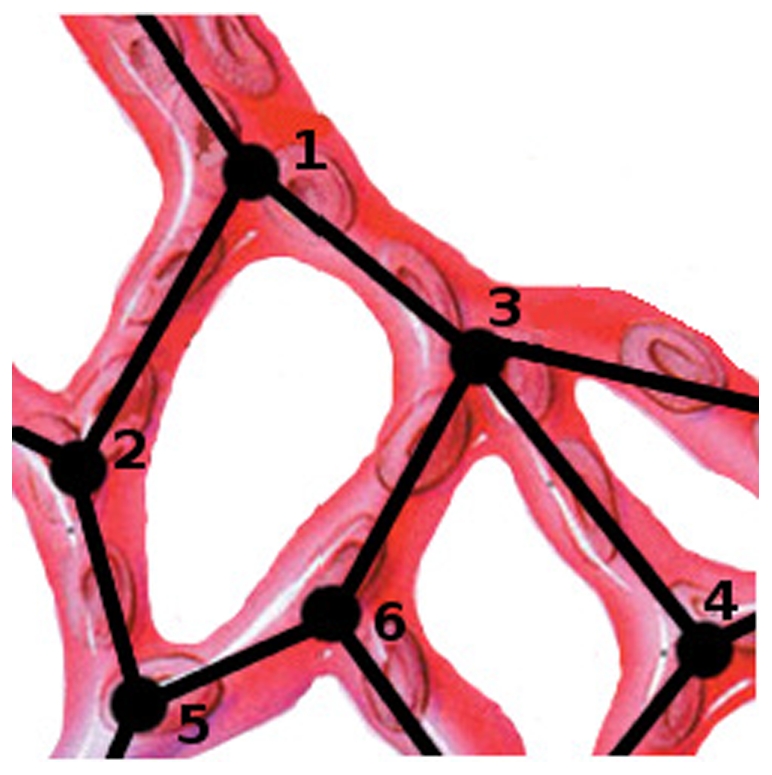
Schematic representation of a vascular graph. A collection of nodes *i* connected by edges *ij*.

The vascular graph model developed by Reichold and coworkers [9] describes flow and transport processes in vascular networks. Here it is used to compute the spatial and temporal distribution of a therapeutic agent in the pulmonary arteries, arterioles, venules and veins: a single-phase two-component (blood and therapeutic agent) scenario. A brief summary of the VGM is given and extensions/adaptations of the vascular graph model are explained (for in-depth information see [9]). The VGM treats the vasculature as a graph, i.e. a collection of vertices or nodes, connected by edges (see Figure 2).

The nodes are the locations at which the vessels bifurcate or end. The edges represent the blood vessels themselves. The diameters of blood vessels vary along their length; typically they are widest at the points of bifurcation. The VGM assigns a mean diameter to each vessel and computes its conductance based on this value. If two adjacent nodes (vertices that are connected by an edge, e.g. node 1 and 2 in Figure 2) are at different blood pressure values, blood flow will be induced between them. For every node 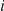 of the vascular graph a continuity equation can be formulated:
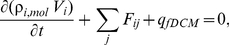
(1)where 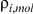, 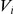 are the blood density and the volume term at node 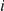, respectively. The mass flow rate through the pulmonary vessel segment 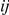 is denoted 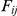 and 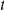 designates the time. To a good approximation, it may be assumed that blood is an incompressible fluid. Therefore, 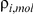 will remain constant unless the blood composition changes significantly. The variable 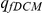 mediates the coupling of mass flow between the double-continuum model and the vascular graph model. In particular, 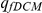 is zero for every node 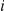 of the vascular graph except for the so-called upscaled nodes (for details see Section 1.3).

The mass flow rate between two nodes 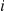 and 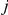 depends on their pressure difference 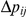, their geometrical distance 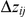 in the direction of gravitational acceleration 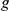 (assumed to act in negative z-direction), the molar fluid density 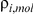, and the vessel resistance 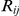:
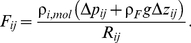
(2)


By inserting (2) into the continuity equation (1) for all vertices, one obtains a linear system of equations whose solution yields the vertex pressures. The flow in the pulmonary vasculature can then be computed from the pressure field using (2). The system of equations is linear due to the fact that the coupling variable 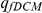 is known at the time of solving the equations of the VGM (see Section 1.3).

**Figure 3:**
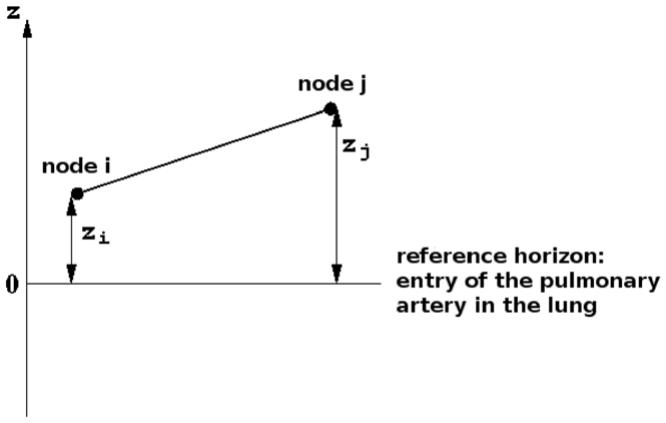
Gravity dependence of pulmonary blood flow.

The distribution of blood in the lung is a function of the cardiac output, gravity, and pulmonary vascular resistance. An average human lung is about 30 cm long from the base (bottom of the lung) to the apex (top of the lung). The pulmonary artery enters each lung about midway between base and apex. Due to the influence of gravity, most of the blood flows through the lower half of the lung [15]. The model captures the gravitational effects, by correcting the blood pressure value of each vertex by 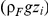, where 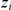 is the distance of node 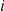 to the entry of the pulmonary artery into the lung (see Figure 3). The difference in 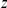 of two nodes 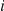 and 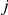 appears as 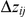 in (2).

The resistance 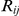 to flow within a pulmonary vessel segment 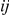 is a result of the viscous forces, of the friction between the flowing blood and the vessel wall, as well as of the friction between the different blood components. Assuming that the flow can be described by the Hagen-Poiseuille law (neglecting wall roughness), the resistance can be written as:
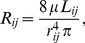
(3)where 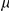 is the dynamic blood viscosity, 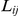 and 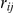 are the length and radius of the vessel segment, respectively. Blood is a heterogeneous, non-Newtonian fluid that exhibits pseudoplastic behavior [16]. The interactions of the different blood components, which are the main origin of the blood properties, are accounted for implicitly via a non-constant viscosity that depends mainly on the vessel diameter and the hematocrit. In-vivo data reported by Lipowsky and coworkers [17] are used to determine the hematocrit in a vessel based on its diameter. In a second step, the blood viscosity within the vessel is computed from the hematocrit value using the relation derived by [18].

The volume 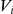 associated to a node 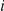 (as in (1)), is defined as the sum of half the volume of all adjacent edges:
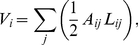
(4)where 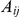 is the cross-sectional area of vessel 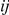. The diameter of the blood vessels, and consequently the vessel volume, change dynamically with the transmural pressure. The VGM takes this effect into account by making the area of the cross-sections pressure dependent. In this work, the values proposed by [19] are used to describe the cross-sectional compliance 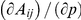 of the different types of pulmonary vessels.

The transport of the dissolved drug molecules with the blood stream is modeled by the subsequent equation:
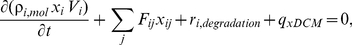
(5)where 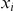 is the mole fraction of the therapeutic agent within the volume of node 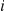, 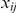 represents the mole fraction of dissolved drug at the physical upstream node and 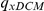 describes the coupling of the double-continuum model to the vascular graph model, taking into account the exchange of dissolved drug molecules between the two models (see Section 1.3). The variable 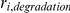 is the sink term that accounts for the degradation processes of the therapeutic agent such as micturition and metabolic transformation reactions [20]:
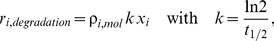
(6)where 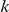 denotes the first order rate constant of a drug administered by a bolus injection and 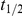 is the half-life of the administered drug.

### 1.2 Alveolus Model - A Double-Continuum Approach

**Figure 4:**
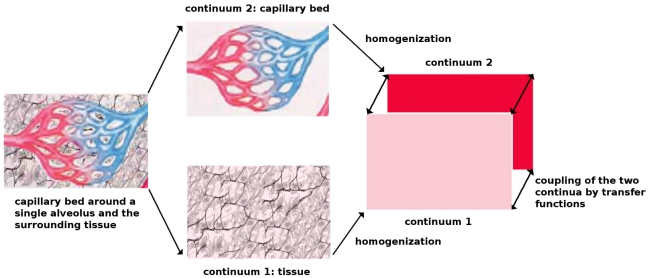
Explanation of the general concept of the double-continuum model.

The events occurring in the arteries, arterioles, veins and venules are described by the vascular graph model. The flow, transport, and reaction processes within the capillaries around a single alveolus and the surrounding tissue are modeled using a double-continuum approach (see Figure 1 and Figure 4). The models of [7] and [5] also comprise a double porous medium using Darcy's law for the flow through the interstitium and the vasculature. However, the alveolus model differs from these previously presented models in that the coupling functions for the flow and transport processes between the capillary bed and the interstitium are the Starling equation (see (18)) and the Stavermann-Kedem-Katchalsky equation (see (19)) respectively. Both equations are traditionally used to describe microvascular liquid transport in the lung [21]. Therefore they are ideally suited as coupling functions for the alveolus model (see Section 1.2.4).

#### 1.2.1 Double-Continuum Model (DCM) - The General Model Concept

There are two main possibilities to describe the flow and transport processes in a biological system: the molecular approach and the continuum approach. The molecular approach considers the movement of single molecules or particles and their interactions under external influences. As the inner diameter of an alveolus is in the order of 140 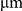, the domain size of the alveolus model is very large compared to the size of fluid and drug molecules. The excessive number of computational particles required thus forbids the usage of a molecular approach. Therefore, a continuum approach is chosen instead, to describe the transport of the dissolved drug molecules through the pulmonary capillaries and tissue, as well as the exchange between compartments.

The structure of the biological system can also be considered either in a discrete or continuous fashion. To make the transition from a discrete to a continuum description, the concept of a representative elementary volume (REV) is used [22]. The pulmonary tissue and the capillary bed are both described as two distinct continua. By means of volume averaging, the discrete properties of the capillary bed and pulmonary tissue (such as the size of the different cell types, pore-space geometry, capillary characteristics) are represented by a continuum with new effective parameters, e.g. porosity, tortuosity or permeability (for methodological details see [23]).

As shown on the left image in Figure 4, the pulmonary capillaries are embedded in the pulmonary tissue. Flow, transport, and reaction processes in both compartments are of interest - they are, however, very different from each other. Therefore, the capillary bed around a single alveolus and the surrounding tissue are treated as two separate continua (see Figure 4). The flow and transport processes between them, i.e. the exchange of fluid and substances across the capillary walls into the pulmonary tissue and vice versa, are honored via exchange terms, the so-called transfer functions (see Section 1.2.4). The tissue continuum consists of cells, fibers, amorphous ground substance and interstitial fluid. The individual components are not densely packed. Therefore, a fraction of the interstitial fluid can flow freely within the tissue. Hence, the pulmonary tissue can be described with a porous medium approach as it has already been done by [24]. About 1800 capillary segments, of 10 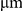 average length and 8 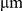 mean diameter, enwrap a human alveolus [14]. The whole lung consists of 300 million alveoli [25]. Therefore, a discrete modeling approach would need to resolve 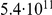 capillary segments. To avoid the high computational expense incurred, one can introduce a capillary continuum instead, which represents the pulmonary capillary bed around one alveolus as an averaged quantity. This porous media concept requires effective parameters to be determined, such as the permeability and porosity. The permeability expresses the ability of a porous medium to transmit fluids. In the case of the capillary continuum, this is determined by the spatial distribution and the cross linking of the individual vessel segments. The Hagen-Poiseuille law is a measure of the blood flow velocity in the vessels [16] and relates it to the permeability (see Section 2.3). The volume averaging over the capillary bed results in a porosity value of one, i.e. the volume of the voids is equal to the total volume.

In summary, the double-continuum approach treats pulmonary tissue and capillary bed as two separate porous media continua. The interactions between them are taken into account by transfer functions.

#### 1.2.2 Pulmonary Tissue Continuum

The phase moving within the tissue continuum consists of two components, namely the interstitial fluid and the therapeutic agent. It is assumed that the fluid phase is incompressible. Thus, the movement of the dissolved drug molecules in the interstitial tissue of the lung is modeled using a single-phase two-component approach in a rigid, porous medium. The influence of the respiratory movement on the pulmonary tissue is not considered. The drug molecules are completely miscible with the interstitial fluid. The interstitial fluid is treated as a Newtonian fluid because it consists mainly of water. It has a composition similar to blood plasma, which consists of 90 percent of water, nine percent organic, and one percent inorganic substances that are dissolved in water [16]. With the additional assumption that the flow within the tissue is creeping, the flow velocity of the interstitial fluid can be described by Darcy's law:
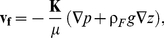
(7)where 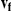 is the Darcy velocity, 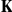 is the intrinsic permeability tensor, 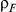 is the mass density of the fluid, 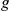 is the gravitational acceleration, and 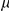 is the dynamic viscosity of the fluid phase. The calculation of the flow velocity through the interstitium with Darcy's law has been done previously, for example, by [2] or [26].

Due to the assumption of an incompressible fluid phase and a constant tissue porosity, the temporal variation of the product of porosity 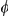 and molar density 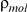 does not have to be considered in the continuity equation. The following form of the continuity equation is used:
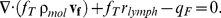
(8)


Here, 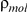 is the molar density of the fluid and 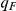 is the coupling variable for the flow between the two continua. The exact definition of the variable 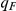 is given in Section 1.2.4. The flow processes in the tissue and capillary continuum are calculated in the same model domain. For this reason, a factor 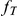 is introduced to describe the volume fraction of tissue within the model domain. The volume fraction of tissue 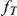 and the capillary volume fraction 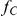 add up to unity:
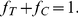
(9)


The exchange of fluid and dissolved components between the tissue and capillary continuum is a surface related process. Therefore, the intercompartmental exchange rate (and thus, the coupling variable 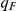) depends, among others, on the surface area of the capillaries per unit volume of tissue 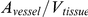 (see (18)). It is therefore not explicitly corrected with the tissue volume fraction 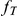.

At the arterial side of the capillary bed, about 0.5 percent of the plasma that flows through the capillaries is filtered out into the surrounding tissue. 90 percent of this extravasated fluid is reabsorbed at the venous side of the capillary bed. The remaining 10 percent of the extravasated fluid is removed by the lymphatic system from the interstitial space [16]. The lymphatic system carries the excess of interstitial fluid, and, with it, dissolved and suspended substances like macromolecules through the lymph vessels and nodes into the great veins [27]. The influence of the lymphatic system on the mass balance (8) is included by the sink term 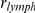 [2]:
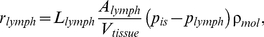
(10)where 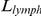 is the hydraulic conductivity of the lymphatic vessel wall, 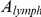 is the surface area of the lymphatic vessels in the lung, 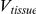 is the unit volume of tissue, 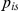 and 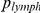 are the hydrostatic pressure in the interstitial space and lymphatic system, respectively. As there is no functional lymphatic system within a tumor [28], the sink term 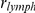 is omitted in tumor tissue.

The transport of the dissolved therapeutic agent in the pulmonary tissue is described by the following equation:
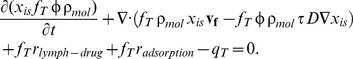
(11)


The first term of (11) is the so-called storage term. It describes the temporal variation of the product of tissue volume fraction 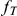, porosity 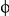, molar density 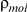 and mole fraction of the dissolved component 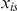. The advective and diffusive transport of the therapeutic agent within the tissue are expressed by the second term. The diffusive transport of the drug depends on the aqueous diffusion coefficient 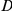 of the therapeutic agent and the tortuosity 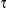 of the tissue. The tortuosity characterizes the degree of sinuousness of the routes of transport within the porous medium. The variable 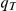 is the transport coupling variable. It defines the amount of dissolved drug molecules that is transported from the intravascular space across the capillary wall into the tissue and vice versa (see Section 1.2.4). The sink term 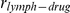 describes the reduction of the number of drug molecules by the lymphatic system:
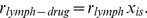
(12)


This sink term is defined in a similar way as the term for the flow reduction by the lymphatic system (see (10)), except that the mole fraction 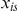 of the dissolved therapeutic agent is required in addition.

The sink term 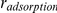 [29] defines the interaction of the drug molecules with the tumor cells. The ligand-receptor interaction decreases the mole fraction of free flowing therapeutic agent:
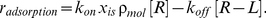
(13)


Here, 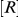 is the receptor concentration, 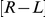 is the receptor-ligand-complex concentration, 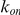 is the kinetic constant for the forward reaction, i.e. the binding of a ligand of the drug molecule with a tumor cell receptor. The kinetic constant for the backward reaction, i.e. the splitting of the chemical bond between the therapeutic agent and the cell, is termed 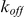.

The sink term 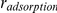 is only considered in tumor regions, whereas 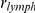 is only included in regions of healthy pulmonary tissue.

#### 1.2.3 Pulmonary Capillary Bed Continuum

The capillary continuum represents the pulmonary capillary bed around one alveolus as an averaged quantity. The movement of the dissolved drug molecules within the pulmonary capillaries is described with a single-phase two-component approach. The incompressible fluid phase consists of the two, completely miscible, components: blood and therapeutic agent. According to [30], the velocity within the single capillaries is constant in time. The capillaries may be treated as rigid tubes [19] and due to the low Reynolds number within the capillaries, about 0.003 according to [31], the flow is creeping. Currently, the double-continuum model does not account for variations in capillary morphology. It assumes that the diameter of all pulmonary capillaries is constant (8 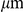: the mean pulmonary capillary diameter according to [14]). Consequently, a constant viscosity value of 0.0021 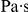 is assumed, which agrees with the diameter and hematocrit dependent viscosity relation developed by [18] that is used in the VGM.

**Figure 5:**
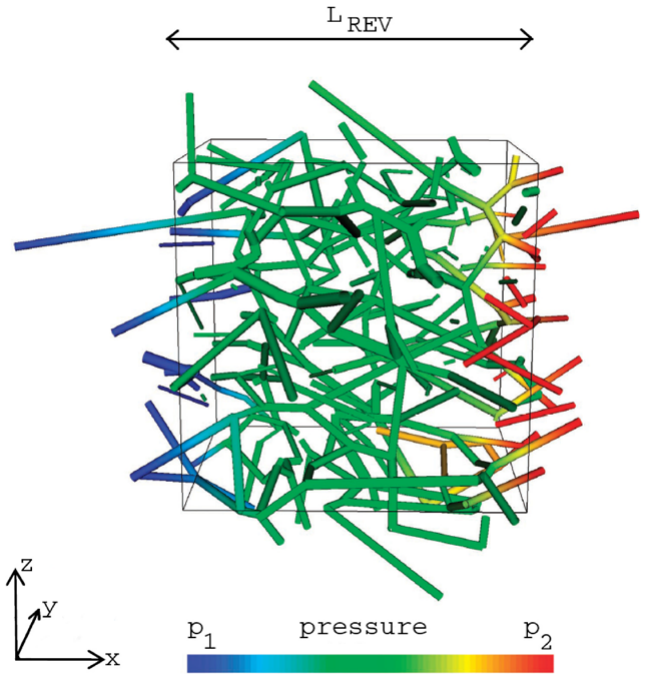
Computation of the effective permeability of a REV. Fixed pressures 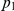 and 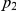 set at all nodes crossing the left and right face normal to x respectively (no-flow boundary condition at all nodes crossing the other four faces). Effective permeability computed from pressure gradient and integral mass flow 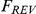 through the REV's capillary network (image modified from [9]).

**Figure 6:**
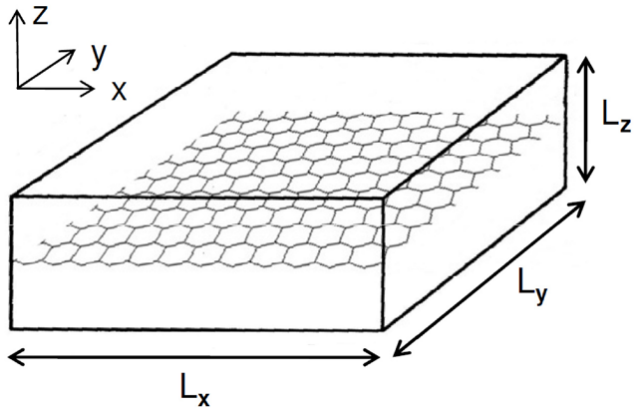
Determination of the permeability tensor for the capillary continuum. Hexagonal mesh of pulmonary capillaries embedded in a cuboid.

As the capillary bed is treated as a porous media continuum, Darcy's law may be applied to determine the blood flow velocity. This has been demonstrated by [32]. The method requires the computation of the continuum's intrinsic permeability tensor, which depends mainly on the connectivity of the capillary segments and their diameter. The intrinsic permeability tensor of the capillary bed can be obtained analogous to the effective conductance computation in [9]. The domain is divided into a number of cuboid subvolumes. In order to compute the permeability of one such subvolume in x-direction, the integral mass flow 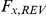 is computed between the two faces normal to the x-axis (using the VGM and the discrete capillary network). Arbitrary (but different) pressure boundary conditions 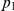 and 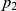 are set at all vessel-endpoints crossing the two respective faces, and no-flow boundary conditions are set at the remaining four faces (see Figure 5). The permeability of the subvolume in x-direction now reads:
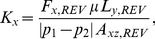
(14)where 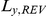 is the length of the cuboid in y-direction and 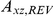 is the cross-section of the considered REV parallel to the x-axis. The permeabilities in y- and z-direction are computed analogously. Repeating this method for each subvolume yields a heterogeneous permeability field for the capillary bed around a single alveolus. Ideally, one would use a high-resolution angiography technique, such as synchrotron radiation X-ray tomographic microscopy (srXTM), to obtain the fully resolved capillary network around an alveolus. Then, the above methodology can be applied to determine a realistic intrinsic permeability tensor. As high-resolution pulmonary angiography data are not available for the present work, an artificial network is constructed instead. According to [33], the alveolar capillaries form a hexagonal network. Using the numerical values provided by [14], an artificial capillary bed is constructed consisting of 1800 capillary segments, each 8 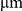 in diameter and 10 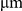 long (see Figure 6). The intrinsic permeability tensor of the pulmonary capillary bed is obtained for the cuboid illustrated in Figure 6 by using the aforementioned method. The dimensions of the cuboid in which the hexagonal network of the capillaries is embedded depend on the size of the model domain of the alveolus model and have an influence on the permeability values (results shown in Section 2.3).

With the assumptions made at the beginning of Section 1.2.3 and given the intrinsic permeability tensor of the capillary continuum, the flow of blood and dissolved therapeutic agent can be described with the following continuity equation:
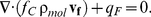
(15)


The transport of the dissolved therapeutic agent is represented by the subsequent equation:
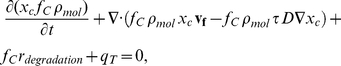
(16)where 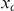 is the mole fraction of drug molecules dissolved in blood. The degradation processes of the therapeutic agent are equal to those described earlier for the vascular graph model, hence 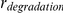 is given by (6). As the porosity 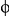 of the capillary continuum is set to one, 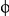 does not appear in (15) and (16).

#### 1.2.4 Coupling Functions for the Flow and Transport Processes between the two Continua

**Figure 7:**
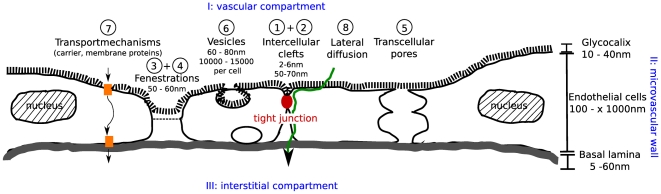
Paracellular and transcellular pathways [48].

The flow and transport processes between tissue and capillary continuum are described by the coupling functions 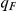 and 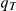, based on the transepithelial transport via transvascular pathways. Figure 7 illustrates the different cell morphologies, which can facilitate transvascular transport, namely interendothelial clefts, fenestrae, transcellular pores and vesicles. There are two main modes of transport: transcellular and paracellular. The transcellular pathway crosses the apical and basolateral membrane of the endothelial cell and in most cases leads through a part of the intercellular cleft. The paracellular way spans the entire length of the intercellular cleft.

**Figure 8:**
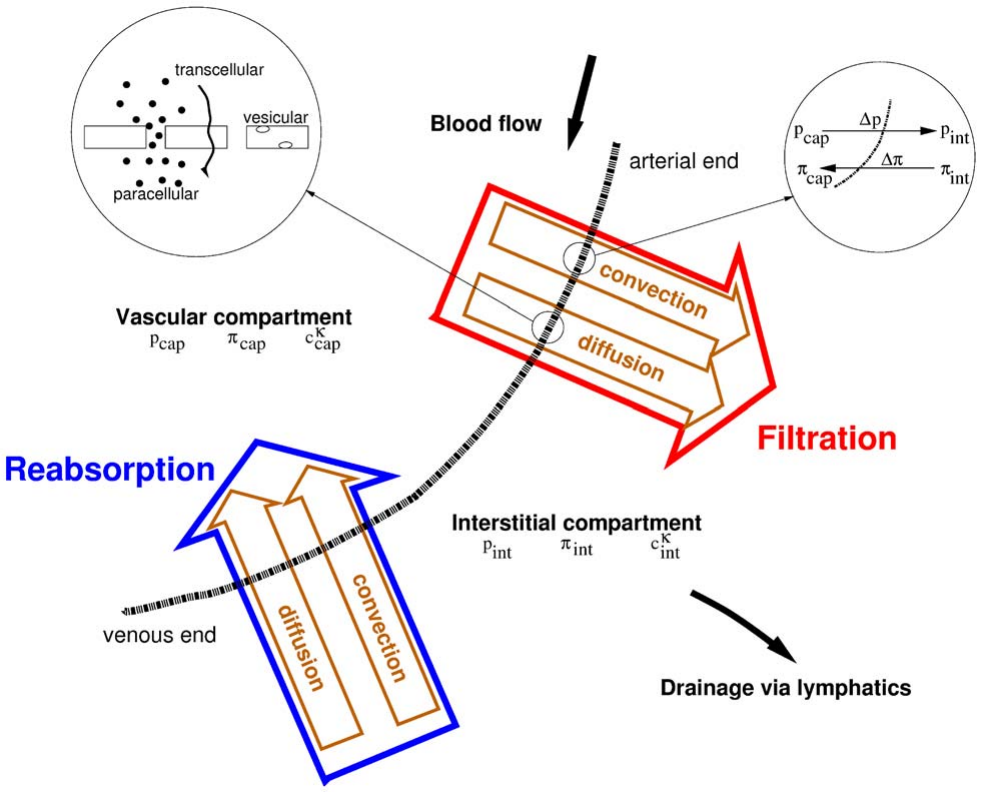
Processes and forces of transvascular exchange [48].

The transport across the capillary wall mainly depends on relative pressure and concentration gradients (see Figure 8). The hydrostatic and the oncotic pressures in the capillary and the interstitial space determine the direction and the magnitude of fluid flow between the two compartments. The oncotic pressure is the sum of the colloid-osmotic pressure and an osmotic pressure caused by the Gibbs-Donnan effect. The colloid-osmotic pressure relates to the osmotic pressure caused by macromolecules. The large anionic proteins in blood plasma cannot pass through the capillary walls. Small cations are attracted, but not bound to the large anionic proteins. Consequently, small anions will cross the capillary walls away from the plasma proteins more rapidly than small cations. This unequal distribution of permeable ions between the intravascular and the interstitial space is called Gibbs-Donnan effect and influences also the flow of water across the semipermeable capillary wall.

The outflow of fluid from the capillaries into the interstitium across the microvascular wall is called filtration or extravasation. The inflow of fluid is termed reabsorption. The extravasated fluid can either be reabsorbed by the same or a different capillary, or it can leave the tissue via the lymphatic system [34]. The difference of the hydrostatic pressures, also called transmural pressure, and the oncotic pressures between the intravascular and the interstitial space determine the fluid transport through the capillary wall:
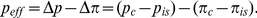
(17)

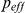
 is the effective filtration pressure, 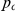 and 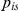 are the hydrostatic pressure in capillaries and interstitial space respectively. 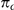 and 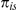 are the corresponding oncotic pressures. Solvent flux across the microvascular wall is proportional to the effective filtration pressure 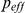. According to Starling's law, net fluid flow across a vessel wall is given by [35]:
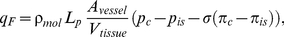
(18)where 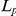 is the hydraulic conductivity of the vessel wall, 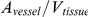 is the surface area of pulmonary capillaries per unit volume of tissue. The capillary wall acts as a semipermeable membrane and thus has a strong influence on the degree of transvascular fluid flow. The reflection coefficient 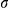 describes how well solute particles can move across the vessel wall. It can vary from zero (i.e. no reflection, all particles pass the barrier) to one (impermeable membrane). Equation (18) is used as the coupling function 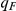 for the fluid flow across the interface between tissue and capillary continuum in the mass balance equations (8) and (15). At the arterial side of the capillary bed, the transmural pressure is higher than the osmotic pressure difference between the plasma and interstitial fluid. Therefore, an outflow of fluid is observed at these locations. Due to the flow resistance in the blood vessels, the hydrostatic pressure in the capillaries decreases along their length. At the venous side of the capillary bed, the transmural pressure is equal to or often smaller than the osmotic pressure difference. This is where reabsorption of water takes place.

The Stavermann-Kedem-Katchalsky equation describes the advective and diffusive transport of the therapeutic agent across the microvascular wall [35]:
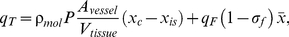
(19)where 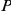 is the permeability of the capillary wall. The solvent-drag reflection coefficient 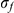 describes the retardation of the therapeutic agent as it passes through the vessel wall. The variable 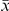 represents the mean mole fraction of dissolved therapeutic agent within the pores of the capillary walls. According to [35], the mean mole fraction of dissolved therapeutic agent within the pores can be calculated by the logarithmic mean: 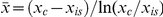. Here, 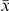 is approximated by 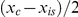. Equation (19) is used as the coupling function 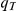 for the transport of the therapeutic agent between tissue and capillary continuum in the component mass balance equations (11) and (16).

### 1.3 Coupling the Alveolus Model to the Vascular Graph Model

**Figure 9:**
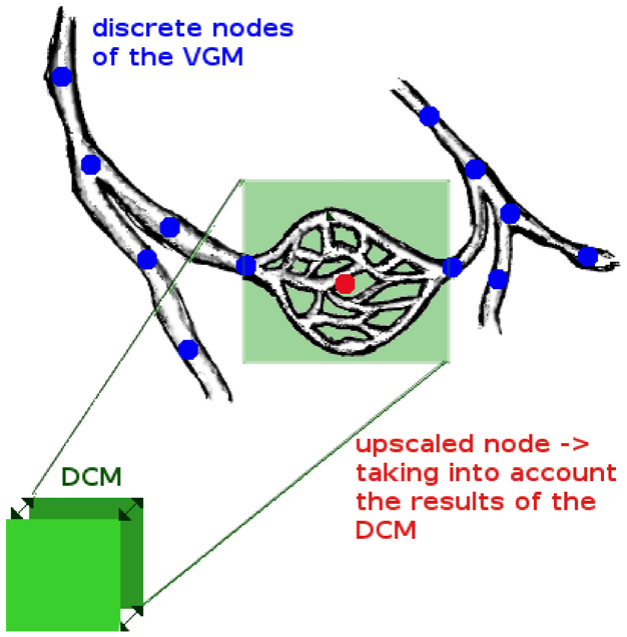
Concept for the coupling of the DCM to the VGM.

The vascular graph model and the alveolus model have been described in Sections 1.1 and 1.2 respectively. This section gives an overview of the approach used to couple the two models. The coupling of VGM and DCM has the advantage that one obtains a discrete representation of the vasculature where it is computationally affordable (i.e. at the non-capillary level) and a continuum representation where a fully-resolved approach would be too expensive (i.e. at the level of the capillary bed and its surrounding tissue). The vascular graph model describes the flow and transport of the therapeutic agent within the non-capillary pulmonary vasculature. Each pre-capillary arteriole and each post-capillary venule are connected via an upscaled edge to a so-called upscaled node, representing the capillary bed of a single alveolus and its associated tissue. These upscaled nodes are in turn described by the double-continuum model. It is at these sites that the administered drugs and blood plasma can leave the blood compartment and enter the surrounding tissue. The double-continuum model is used to compute the amount of therapeutic agent and fluid leaving the blood stream and provides this information to the VGM, where it is incorporated as additional sink terms 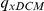 and 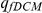 for the upscaled nodes. This coupling concept is illustrated in Figure 9. At each time step, the pressure and the flow field for the whole vascular graph and the distribution of the therapeutic agent in the graph are computed. Then, the additional sink terms, also termed correction factors, are recomputed separately for each upscaled node of the vascular graph by calling the double-continuum model with the Python subprocess module. The correction factors correspond to the amount of therapeutic agent and fluid that leave the blood compartment in a time 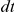, which is the duration of an (explicit) time step of the VGM transport simulation. After the system of equations of the DCM has been solved for every upscaled node of the VGM to obtain the two coupling variables 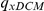 and 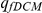 for the current time step of the VGM, the pressure and the flow field of the vascular graph and the transport of the drug through the graph are corrected by the calculated sink terms. Thus, the DCM and the VGM are linked using sequential execution and data transfer through the coupling variables 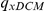 and 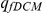. Initial and boundary conditions of the pressure, as well as the mole fraction of the dissolved therapeutic agent are required for both continua of the alveolus model, in order to solve for the additional sink terms 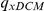 and 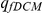. The pressure boundary conditions for the capillary continuum are taken from the two VGM nodes adjacent to the upscaled node. As the VGM provides no information about pressure in the pulmonary tissue, the pressure boundary condition for the tissue continuum is taken from literature (see [34] and [28]). The two VGM nodes on the arterial and on the venous side of the capillary bed provide the initial condition of the therapeutic agent concentration in the capillary bed. A linear relationship for the initial concentration in the capillary bed continuum between the two nodes connected to the upscaled node is taken. The initial drug concentration in the tissue is assumed to be zero. At every further time step, the final concentration of the previous time step is taken as initial condition.

## Results

**Figure 10:**
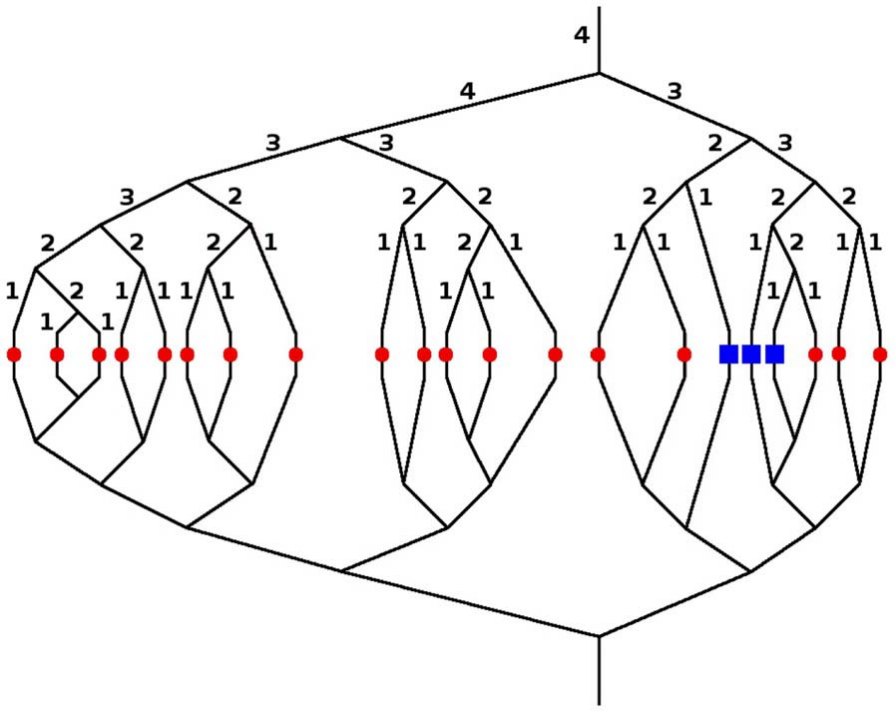
Visualization of the vascular graph coupled to the DCM. The black lines represent the blood vessels, which form the unstructured grid of the VGM. The red nodes and the blue rectangles symbolize the capillary bed and pulmonary tissue around an alveolus, which are simulated using the DCM. The red nodes are the healthy upscaled nodes and the blue rectangles are the tumorous ones. The blood vessels above/below the alveoli are arteries/veins of the order one to four, with a morphology according to Table 1 and Table 2 respectively. The numbers indicate the classification of the vessel segments according to the Strahler ordering system.

This section demonstrates how the coupled model described in Section 1.3 can be applied to simulate therapeutic agent kinetics in the lung. The computational grid is based on the literature values given in [36] (see Table 1) and [37] (see Table 2). Horsfield and coworkers studied the morphometry of the human pulmonary arterial and venous trees, based on Strahler's ordering system. The Strahler algorithm defines the smallest non-capillary blood vessels as order 1. If two vessels of the same order meet, the order number of the confluent vessel will be increased by one. If a blood vessel of order n meets another vessel of an order smaller than n, the order of the confluent vessel will remain n ([38], see also Figure 10). The precondition that the Strahler ordering system can be used for the classification of pulmonary vessels is the dichotomous branching of the pulmonary vascular tree [25].

**Table 1:** Model of the pulmonary arterial tree (according to [36]).

order	number of branches	diameter in mm	length in mm
17	1.000	30.000	90.50
16	3.000	14.830	32.00
15	8.000	8.060	10.90
14	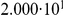	5.820	20.70
13	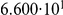	3.650	17.90
12	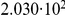	2.090	10.50
11	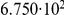	1.330	6.60
10	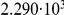	0.850	4.69
9	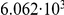	0.525	3.16
8	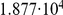	0.351	2.10
7	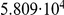	0.224	1.38
6	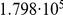	0.138	0.91
5	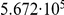	0.086	0.65
4	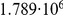	0.054	0.44
3	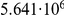	0.034	0.29
2	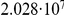	0.021	0.20
1	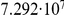	0.013	0.13

**Table 2:** Model of the pulmonary venous tree (according to [37]).

order	number of branches	diameter in mm	length in mm
15	4.000	13.88	36.7
14	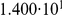	5.23	39.0
13	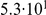	2.90	25.4
12	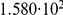	1.90	18.5
11	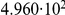	1.21	11.0
10	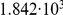	0.61	3.20
9	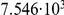	0.39	2,54
8	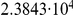	0.22	1.98
7	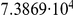	0.14	1.34
6	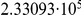	0.096	0.910
5	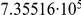	0.064	0.617
4	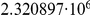	0.043	0.418
3	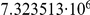	0.029	0.283
2	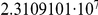	0.019	0.192
1	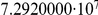	0.013	0.130

### 2.1 Simulation Set-Up

A subset of the full pulmonary vasculature is chosen as the domain for the simulation. Starting from Strahler order 4, a dichotomous branching tree of arterioles is generated, leading to 21 pre-capillary arteries of order 1. These connect to as many upscaled nodes, which in turn are connected to 21 post-capillary venules of order 1. The veins dichotomously reunite until order 4 is reached (see Figure 10).

**Figure 11:**
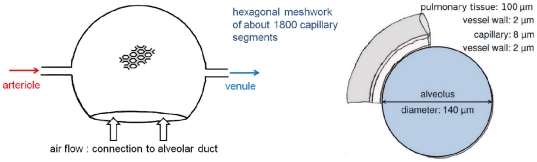
**Left.** Model domain of the alveolus model for an healthy upscaled node consisting of a spherical shell with an inner diameter of 140 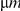 and an outer diameter of 364 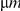 (image modified from Weibel (1991) [33]). **Right.** Different kind of structures comprised by the model domain of the DCM.

The diameters and lengths of the individual vessels are assigned according to the measurement results of [36] and [37], as listed in Tables 1 and Table 2. Three of the 21 upscaled nodes have been assigned tumor properties, representing an alveolar cell carcinoma. These are the three upscaled nodes depicted by the blue rectangles in Figure 10. The model domain of the DCM for a healthy upscaled node represents an alveolus with its capillary bed and surrounding tissue. It is constructed as a spherical shell with an inner diameter of 140 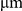 (the mean diameter of a human alveolus according to [14]) and an outer diameter of 364 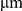 (see Figure 11). On the alveolar surface, the capillaries are arranged in form of a hexagonal network with a few interspersed pentagonal meshes to close the spherical surface [33]. In-between capillary segments and above the capillary mesh, the pulmonary tissue is located. The thickness of the tissue layer, 100 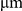, is arbitrarily chosen. The lumen of a pulmonary capillary is about 8 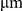 wide, and the endothelial cell wall is approximately 2 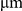 thick (according to [14] and [16]). The individual layers thus add up to a total thickness of 112 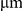. Therefore, the model domain has an outer diameter of 364 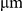. The model domain of the DCM for a tumorous upscaled node is a sphere with a diameter of 364 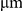. It is assumed that the cancer cells have destroyed the alveolar walls, and have penetrated into and filled the alveolus. The tumor vasculature consists of vessels from the already existing network of the host vasculature and new vessels resulting from the angiogenesis response of the host vessels to the cancer cells [28]. There are no necrotic regions. It is assumed that the arterial input to the capillary bed is on the left side of the alveolus, and the venous drainage occurs at the right side (see Figure 11). Blood cannot enter or leave the model domain via any other pathway. According to [39], the pulmonary arterioles, also termed pre-capillary vessels, give rise to the capillary networks and they number between one and two per alveolus. Therefore, the assumption of a single feeding arteriole and a single draining venule is made.

This setup is meant to demonstrate the functionality of the coupled model rather than being a realistic description of the spatial and temporal distribution of a therapeutic agent within the human lung for cancer therapy. Two simplifications are made. First, the cross-sectional compliance, the pressure dependent change of the cross-section of a vessel segment, is not considered in this example. Second, the influence of the gravity on the blood flow through the vessel segments is neglected. The data of [36] and [37] only allow the generation of a two-dimensional graph. The distance of each single vessel segment to the entrance of the pulmonary artery into the lung cannot be determined.

### 2.2 Initial and Boundary Conditions

The system of equations of the vascular graph model and the double-continuum model are solved for two primary variables: the pressure 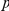 and the mole fraction of dissolved therapeutic agent 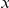. Initial and Dirichlet pressure boundary conditions are set at the root arterial and root venous vertex. The numerical values chosen are 1064 and 199.5 Pa respectively, taken from [15]. At the root arterial vertex, the mole fraction of dissolved therapeutic agent 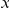 is set to 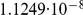. The initial and boundary conditions for the upscaled nodes, where the double-continuum model's system of equations is solved, are automatically taken from the corresponding adjacent VGM nodes, as is explained in Section 1.3.

### 2.3 Parameters

The results, shown in Section 2.4, are based on the parameter values presented here. The vascular graph model requires the diameters and lengths of the vessel segments that make up the vascular graph. The numerical values of these vessel properties are taken from Table 1 and Table 2. Table 3 gives an overview of the parameters used in the alveolus model for the healthy and the tumorous case. Most variables are taken from literature. However, for some parameters no suitable values could be found. For most parameters, Table 3 shows the ranges of the used parameters given in literature.

**Table 3:** Model parameters of the alveolus model.

parameter	symbol	value	parameter range
tissue continuum			
diffusion coefficient^3^	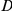 [ 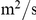 ]	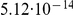 [see text]	-
dynamic viscosity^1^	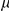 [ 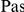 ]	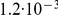 [49]	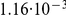 – 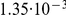 [50]
hydraulic conductivity of lymphatic vessel wall^2^	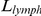 [ 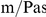 ]	n: 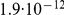 [see text]	-
initial receptor concentration	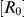 [ 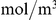 ]	t: 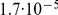 [see text]	-
kinetic constant: forward reaction	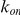 [ 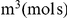 ]	t: 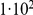 [see text]	-
kinetic constant: backward reaction	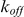 [ 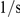 ]	t: 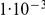 [see text]	-
interstitial fluid pressure	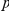 [ 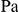 ]	n: −1064 [34]	-
		t: 133	133–3591 [28]
lymphatic pressure^2^	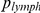 [ 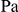 ]	n: −1200[see text]	-
mass density^1^	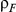 [ 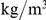 ]	1030 [51]	-
molar density^1^	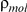 [ 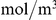 ]	303.5 [16]	-
permeability	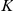 [ 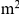 ]	n: 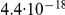	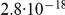 – 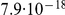 [52]
		t: 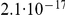	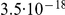 [52]– 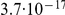 [2]
porosity	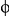 [-]	n: 0.13	0.13–0.3 [2]
		t: 0.27	0.21–0.37 [53]
surface area of lymph vessels per unit volume of tissue^2^	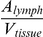 [ 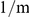 ]	n: 3.0 [see text]	-
tortuosity	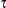 [-]	n: 0.28 [54]	-
		t: 0.71	0.60–0.84 [55]
volume fraction of tissue	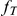 [-]	n: 0.9[see text]	-
		t: 0.8	0.80–0.99 [2]
capillary continuum			
capillary volume fraction	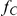 [-]	n: 0.1[see text]	-
		t: 0.2	0.01–0.20 [2]
diffusion coefficient^3^	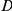 [ 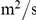 ]	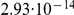 [see text]	-
dynamic viscosity^1^	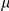 [ 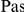 ]	0.0021 [18]	-
half-life of therapeutic agent	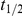 [ 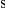 ]	21600[see text]	-
mass density^1^	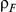 [ 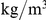 ]	1050	1040–1060 [31]
molar density^1^	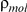 [ 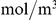 ]	284 [16]	-
permeability	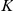 [ 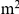 ]	n: see Table 4	-
		t: 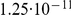 [see text]	-
porosity^3^	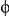 [-]	1[see text]	-
tortuosity^3^	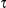 [-]	1[see text]	-
transfer equations			
capillary oncotic pressure	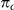 [ 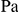 ]	n: 3724 [34]	-
		t: 2660 [2], [43]	-
diffusive permeability	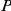 [ 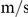 ]	n: 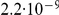	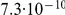 – 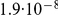 [2]
		t: 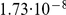	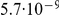 – 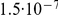 [2]
hydraulic conductivity	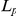 [ 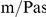 ]	n: 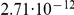 [2]	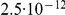 – 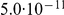 [56]
		t: 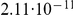 [2]	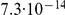 – 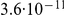 [57]
interstitial oncotic pressure	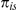 [ 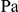 ]	n: 1862 [34]	-
		t: 1995 [2]	-
molar density^1^	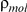 [ 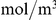 ]	293.75[see text]	-
osmotic reflection coefficient^3^	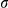 [-]	0.8 [42]	-
solvent-drag reflection coefficient	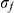 [-]	n: 0.91 [2]	-
		t: 0.82 [2]	-
surface area of capillaries per unit volume of tissue	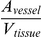 [ 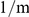 ]	n: 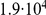 [see text]	-
		t: 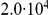 [2]	-

1 Fluid properties do not change in a tumor.

2 There is no lymphatic system in a tumor.

3 The healthy parameter value is also taken for the tumor area.

t: tumor tissue; n: normal tissue.

As it has already been discussed in Section 1.2.3, the processes in the alveolar capillary bed are described with a porous media approach. The results of the permeability field calculation for a healthy upscaled node are shown in Table 4. The cuboid used to compute the permeability tensor has an edge length of 364 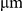 in x- and y-direction and extends 112 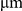 in z-direction. The dimensions of the cuboid depend on the model domain of the DCM - a spherical shell with an inner diameter of 140 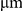 and an outer diameter of 364 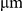. The permeability in z-direction is zero because the capillary bed forms a two-dimensional hexagonal mesh in the xy-plane (see Figure 6). As the capillary bed enwraps the spherical alveolus, it is necessary to transform the permeability tensor computed for the cuboid 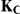 into the coordinate system of the model domain, a spherical shell, to obtain the permeability tensor of the capillary bed 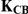. This is done by setting:
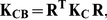
(20)where 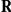 is the three-dimensional rotation matrix and 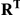 is its transpose. The result of this transformation is the symmetric, positive semidefinite matrix 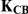. Due to the transformation of the permeability tensor from the coordinate system of the cuboid to the coordinate system of the spherical shell, the permeability values normal to the surface of the shell become zero. Solid tumors are characterized by many tortuous vessels, shunts, vascular loops, irregular intervascular distances and large avascular areas [40]. Hence, the capillary bed for the three tumorous upscaled nodes is not represented by a hexagonal meshwork. Due to the lack of high resolution angiography data of alveolar cell carcinoma, the Hagen-Poiseuille equation is used to estimate the permeability **K** of the tumorous capillary bed. Comparing the Hagen-Poiseuille equation with Darcy's law (see (7)), a relationship for the permeability **K** of the capillary bed of the tumor can be found:
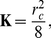
(21)where 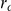 is the mean capillary radius of a tumor vessel. According to [41], 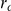 is set to 10 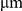. The diffusion coefficient 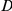 of the injected therapeutic agent depends strongly on the size of the considered drug molecules. For drug molecules dissolved in blood and interstitial fluid, it can be calculated using the Stokes-Einstein-Equation:
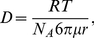
(22)where 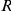 is the gas constant and 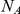 is the Avogadro number. For the body temperature 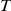 a value of 310.15 Kelvin is assumed. The radius 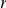 of the drug molecules is approximated by a mean value of 3.7 nm. The value for the dynamic viscosity 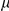 of blood and of the interstitial fluid are assigned as listed in Table 3. The balance equations (8) and (11) of the tissue continuum contain a sink term that considers the influence of the lymphatic system on the amount of interstitial fluid and drug molecules within the tissue. Lymph formation occurs when the lymphatic pressure 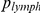 drops below the pressure in the interstitial space 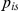 (see (10) and (12)). However, the pulmonary lymphatic pressure has not yet been measured. Experiments have merely shown that the pulmonary lymphatic pressure is at least as low as the local interstitial pressure [42]. The values for 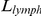, 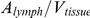 and 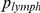 are based on our assumptions. In the case of a tumorous upscaled node, the transport equations of the tissue continuum contain a sink term that considers the interaction of the drug molecules with the cancer cells. The values for the two kinetic constants 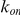 and 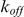 and for the concentration of initially free receptors on the cell surface 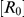 are based on experimental data studying the binding effect of scFv225scTRAIL fusion proteins to H460 cells (unpublished data of M. Doszczak and P. Scheurich, Institute of Cell Biology and Immunology - University of Stuttgart). For the half-life of the drug molecules 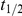, an arbitrary value is used. If the model is applied to a specific drug administered by bolus injection, the properties of that particular therapeutic agent need to be implemented. Further, no specific value for the molar density of blood could be found. The value listed in Table 3 is only valid for blood plasma. The values for the porosity 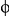 and the tortuosity 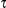 of the capillary continuum are based on the considerations in Section 1.2.3. In case of a healthy upscaled node, the chosen values for the volume fraction of tissue 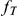 and for the capillary volume fraction 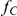 of the model domain are chosen as follows. According to [43], the vascular volume fraction in a 12 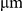 thick section of the alveolar surface is 0.9. Above this layer, we assume a 100 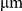 thick layer of pulmonary tissue that is not vascularized. Therefore, the overall volume fractions in the entire model domain (representing a healthy upscaled node) are 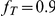 and 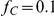 for tissue and capillaries respectively. The transfer equations that couple flow and transport processes between tissue and capillary continuum contain the molar density of the exchanged fluid. In the tissue continuum the considered fluid is the interstitial fluid and in the capillary continuum it is blood. Therefore, the molar density used in the transfer equations is the arithmetic mean of the molar densities of these two fluids. Additionally, the capillary surface area per unit volume of tissue 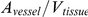 is included in the transfer equations. For the healthy upscaled nodes, this value is calculated based on the geometry of the model domain (a spherical shell):
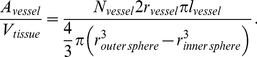
(23)

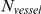
 stands for the number of vessels inside the model domain. 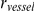 and 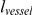 are the radius and length of the capillary segments. 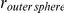 and 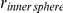 are the radius of the outer and inner sphere respectively.

**Table 4:** Calculation of permeability field of the cuboid shown in Figure 6.

	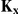 [ 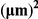 ]	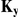 [ 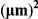 ]	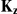 [ 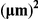 ]
cuboid with hexagonal network	0.15	0.22	0.00

### 2.4 Simulating the Flow and Transport Processes of a Therapeutic Agent within the Lung

The spatio-temporal distribution of a therapeutic agent in the lung is studied numerically using a subset of the full pulmonary vasculature (see Figure 10). The linear system of equations of the VGM is numerically solved using a finite difference method for the spatial discretization and an explicit Euler scheme for the time discretization. The system of equations of the VGM is solved for the two primary variables, the pressure 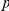 and the mole fraction of dissolved therapeutic agent 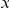, using a decoupled scheme. First, Equation (1) is used to compute the pressure field in the vascular graph. Then, the distribution of the dissolved drug can be determined using Equation (5). As a consequence of the explicit time discretization, the Courant-Friedrichs-Lewy (CFL) condition is applied to guarantee the stability and the convergence of the solution. The transport equation (5) is a hyperbolic partial differential equation. Therefore, a first-order upwind scheme is applied for the discretization of the advection term. The vascular graph model is programmed in Python, performance-critical parts are written as C-extensions using Cython. The coupling of the DCM to the VGM is realized with the Python subprocess module.

The non-linear system of equations of the DCM is numerically solved using a fully upwind vertex centered finite volume method, also called fully upwind box method (see [44]), for the spatial discretization and an implicit Euler scheme for the time discretization. The system of equations of the alveolus model is solved for the four primary variables, the pressures 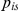 and 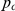 and the mole fraction of dissolved therapeutic agent in the tissue continuum 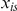 and in the capillary continuum 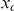, using a fully coupled scheme. Equation (8) and Equation (15) are used to compute the pressure fields in the pulmonary tissue and in the capillaries. Then, the distribution of the dissolved drug in the two continua can be determined using Equation (11) and Equation 16). The coupling of the two continua is realized by the exchange terms 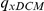 and 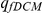 and numerically implemented as additional source/sink terms. The numerical model of the alveolus model is implemented into the open-source porous media simulator DuMu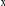, [45], which is based on the Distributed and Unified Numerics Environment DUNE.

The simulation is performed as detailed in the previous sections. Initially, the pressure and flow fields of the vascular graph are computed. Then, a therapeutic agent is introduced at the arterial root vertex. The dissolved drug molecules are advected through the vasculature. At the alveoli, a fraction of the blood plasma and therapeutic agent migrate into the tissue. The exchange rates and phamacokinetics of this process differ between healthy and tumorous alveoli. Due to the intercompartmental exchange, the pressure and flow field of the vascular graph have to be recomputed at each time step.

**Figure 12:**
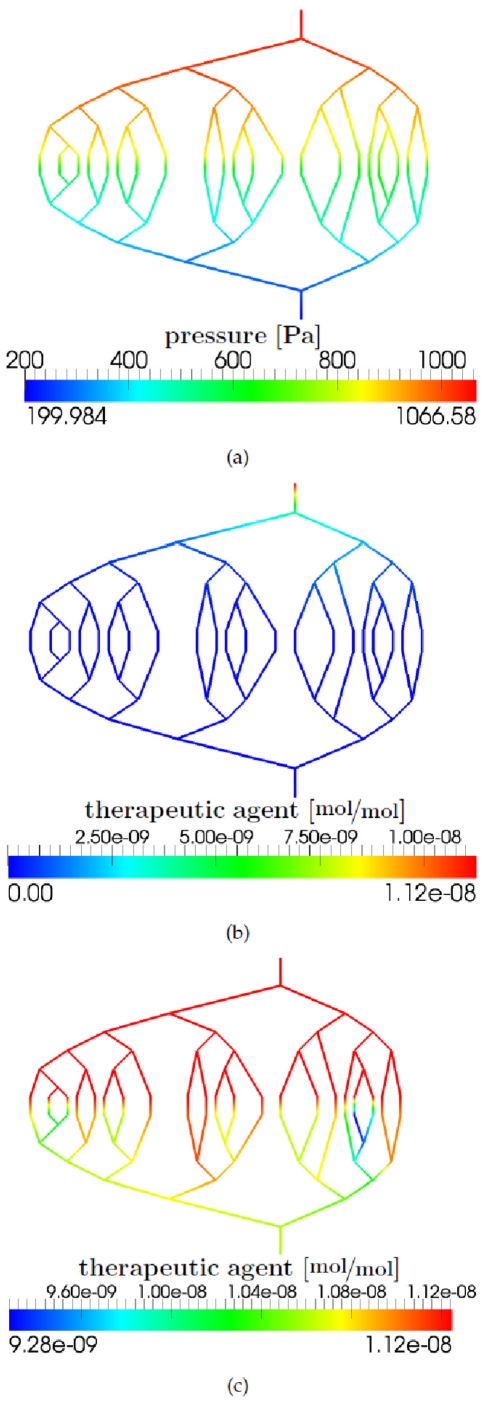
Results of the vascular graph model. (a) Pressure distribution [Pa]. (b) A therapeutic agent is introduced at the arterial root vertex. Drug distribution [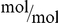] after one time step. (c) Drug distribution [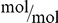] after 11 seconds.

**Figure 13:**
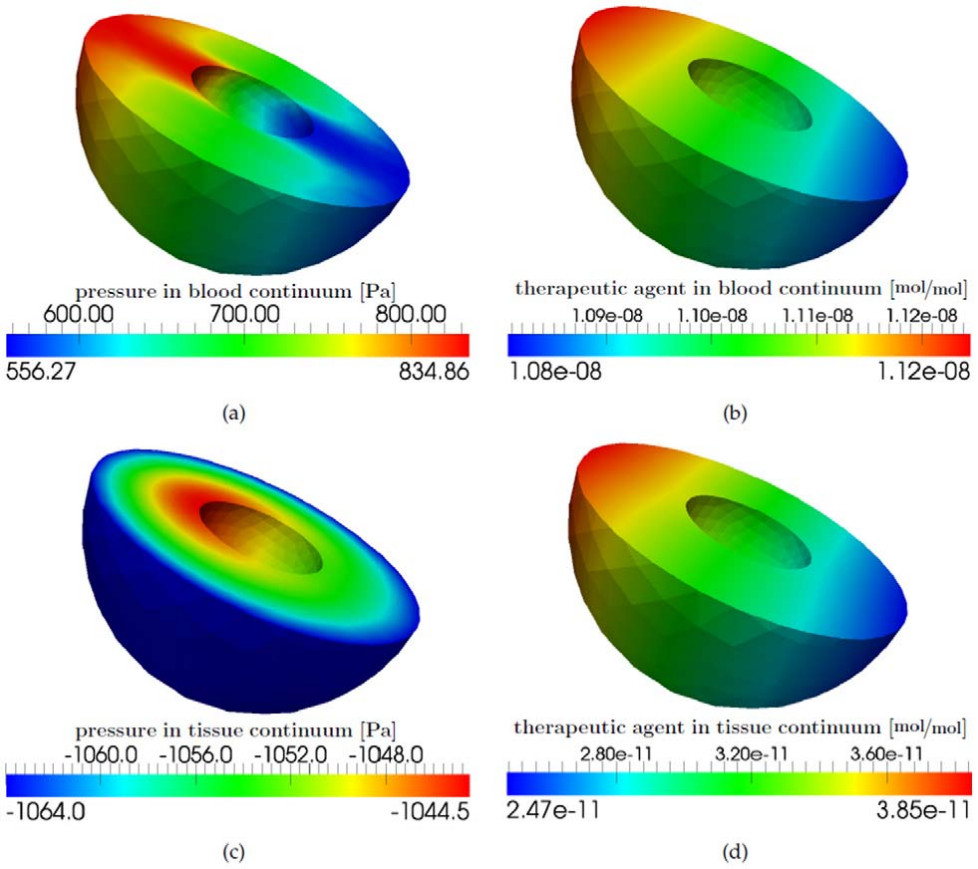
Results for a healthy alveolus (leftmost red node in Figure 10), at the final time step of the simulation. (a) Pressure distribution within the pulmonary capillary bed continuum [Pa]. (b) Drug distribution [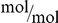] within the pulmonary capillary bed continuum. (c) Pressure distribution within the pulmonary tissue continuum [Pa]. (d) Drug distribution [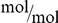] within the pulmonary tissue continuum.

**Figure 14:**
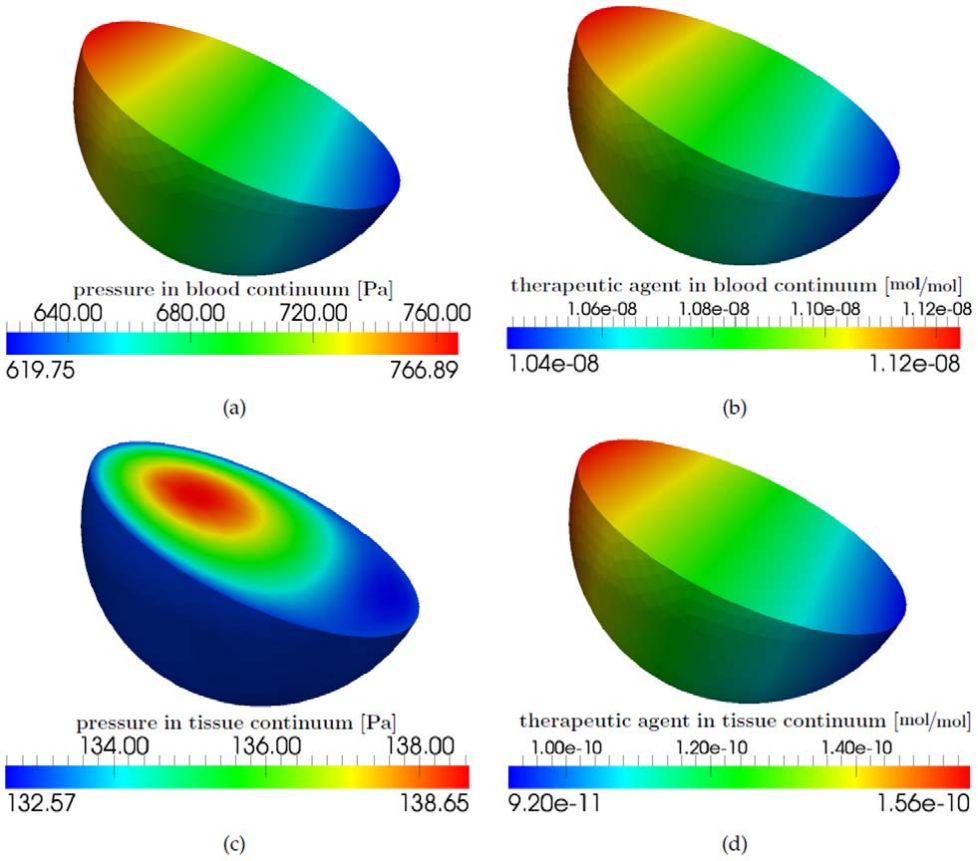
Results for a tumor alveolus (middle blue rectangle in Figure 10), at the final time step of the simulation. (a) Pressure distribution within the pulmonary capillary bed continuum [Pa]. (b) Drug distribution [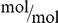] within the pulmonary capillary bed continuum. (c) Pressure distribution within the pulmonary tissue continuum [Pa]. (d) Drug distribution [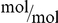] within the pulmonary tissue continuum.

Figure 12 depicts the pressure field and the concentration of the drug molecules at different times during the simulation. The two vessel segments with a Strahler order of 4 have the smallest vessel resistance and therefore the pressure drop is lowest along these segments of the graph (see Figure 12 (a)). In the vessel segments with a Strahler order of 1, the vessel resistance is largest and thus the pressure drop is highest. The effective vessel resistance of a tumorous upscaled node is smaller than the vessel resistance of a healthy upscaled node. Therefore, the pressure gradient along the two edges that are connected with a tumorous upscaled node is smaller (see Figure 12 (a)). The dissolved therapeutic agent is irregularly distributed in the vascular graph (see Figure 12 (c)). The total amount of therapeutic agent that leaves the blood stream at the tumorous upscaled nodes is higher compared to the healthy upscaled nodes (see Figure 12 (c)). The maximum value for the drug concentration in the tissue is also higher for a tumorous upscaled node than for a healthy one (compare Figure 13 (d) with Figure 14 (d)). This depends on the different pressure fields in the blood and tissue continuum for the two kinds of upscaled nodes as well as on the higher diffusive permeability and hydraulic conductivity of tumor vessels and the increased number of blood vessels per unit volume of tissue in the tumor region.

Figure 13 depicts the pressure and mole fraction distribution within the two continua of the double-continuum model for a single healthy upscaled node. For the visualization of the results of a healthy upscaled node, the red node situated in the leftmost position in Figure 10 is chosen. Figure 13 (a) and (b) show a cross-section through the model domain representing the pressure and mole fraction distribution for the capillary continuum. The pressures at the left side and the right side of the capillary continuum are equal to the pressures at the two adjacent nodes connected to the upscaled node. The administered therapeutic agent is distributed within the capillary bed as shown in Figure 13 (b). Figure 13 (c) and (d) show a cross-section through the model domain representing the pressure and mole fraction distribution for the pulmonary tissue continuum. According to [34], the interstitial fluid pressure in the human pulmonary tissue is −1064 Pa. Due to filtration and reabsorption processes across the capillary walls, the pressure slightly changes within the model domain (see Figure 13 (c)). The therapeutic agent is distributed within the pulmonary tissue corresponding to the pressure gradients in the pulmonary tissue. However, the amount of active ingredient is about three orders smaller compared to the capillary continuum (see Figure 13 (d)).

Figure 14 depicts the pressure and mole fraction distribution within the two continua of the double-continuum model for a tumorous upscaled node. In Figure 14 the results of the middle tumorous upscaled node are shown. For the tumorous upscaled node other parameters are used than for the healthy upscaled node (see Table 3). A further difference is that the flow and transport equations of the DCM for the tumorous upscaled node have no sink terms for describing the effects of the lymphatic system. The sink term 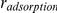 in (11) is only considered for the tumorous case. Due to the pressure distribution in the vascular graph (see Figure 12 (a)), the pressure boundary conditions for the capillary bed continuum of the healthy and the tumorous upscaled node are not the same (see Figure 13 (a) and Figure 14 (a)). The differing permeability fields used for the two kinds of upscaled nodes are a further reason for the different pressure fields in the capillary bed continuum of the tumorous upscaled node and the healthy upscaled node. The administered therapeutic agent is distributed within the capillary bed as shown in Figure 14 (b). Figure 14 (c) and (d) show a cross-section through the model domain representing the pressure and mole fraction distribution for the tumorous pulmonary tissue continuum. According to [28], tumors exhibit high interstitial fluid pressures. Therefore, a Dirichlet pressure boundary condition of 133 Pa is set for the tissue continuum. Due to the lack of a functional lymphatic system and a higher filtration of fluid from the capillaries into the tumor tissue, the interstitial fluid pressure increases further in the model domain (see Figure 14 (c)). However, a certain amount of fluid is reabsorbed at the venous ends of the capillaries. Due to the higher vascular permeability and hydraulic conductivity of tumors [28] and the presence of blood vessels in the whole model domain, the drug molecules are spread in the entire tumor tissue (see Figure 14 (d)).

## Discussion

The model presented in this work describes the flow, transport and reaction processes of a therapeutic agent in the pulmonary circulation, and in healthy, as well as tumorous pulmonary tissue. It accounts for the influence of micturition and metabolic transformation reactions on the agent concentration. Moreover, the role of the lymphatic system as well as the binding of the drug molecules to tumor cells are captured. As such, the model can predict the distribution of a drug administered by continuous bolus injection for the therapy of alveolar cell carcinoma.

In order to guide cancer-therapeutic strategies, however, several important extensions need to be made. The reaction of cancer cells to therapeutic agent binding, the proapoptotic signaling cascade, and the interactions between the individual tumor cells have to be modeled in addition. The model consists of two interconnected sub-models, namely the vascular graph model that describes the processes occurring at the non-capillary level, and the alveolus model that simulates the processes within the alveolar capillary bed and tissue (both in the healthy and disease state). The focus of the model is on predicting the spatiotemporal distribution of therapeutic agents. Angiogenesis and tumor growth are currently not considered. However, these effects occur at timescales that are much larger than those of drug transport and adsorption [2]. Therefore, one can use the model as described in this manuscript to compute the evolution of blood flow and drug distribution as a succession of steady states. After several hours, the vascular and the tissue configuration in the cancerous region change slightly, according to the progression of tumor growth and angiogenesis, which remain to be implemented. The numerical simulation concept presented here, is a first step towards a predictive mathematical and numerical model suitable to guide pulmonary cancer-therapeutic strategies. The concept is chiefly based on theoretical considerations and thus requires validation by comparison with experimental results. Future work will include the acquisition of high-resolution angiography data of the pulmonary circulation (e.g. via srXTM) to generate a realistic vascular graph. A fully resolved capillary bed around a single alveolus and of the alveolar cell carcinoma are the prerequisite to determining the accurate permeability tensor of the capillary continuum in the case of a healthy or a tumorous upscaled node. Once these data are available, the simulation results can be compared with existing measurements to verify the developed numerical model.

As blood is a heterogeneous, non-Newtonian fluid that exhibits pseudoplastic behavior, the VGM determines the blood viscosity within the vessel segments of the considered vascular graph from the hematocrit value using the relation derived by [18]. Currently, the DCM does not account for variations in the capillary morphology. It is assumed that all capillaries have the same physical properties. Consequently, a constant viscosity value is taken. According to [46], models treating capillaries as a spatial continuum with a given permeability which is based on the local morphology of the capillary network neglect the role of the red blood cells on the flow and transport processes through this network of capillaries. As it is already stated in the paragraph above, high resolution angiography data are required for the pulmonary capillary bed and the alveolar cell carcinoma to determine the intrinsic permeability tensor for Darcy's law. The approach for determining the intrinsic permeability tensor from high resolution angiography data should be extended by the continuum model for red blood cell transport in capillary networks presented in [46] and [47] to account for the influence of the red blood cells on the vessel resistance of capillaries.

Until now, the simplifying assumption of a non-pulsating flow through the vessel segments of the vascular graph and the capillary bed of the alveolus model is made. However, the arterial blood pressure rises and falls due to the phases of the cardiac cycle [15]. In the future, the vascular graph model and the double-continuum model should include the cyclic pressure changes in the blood vessels.

It is our goal to further improve the accuracy of the model by the extensions outlined above such that the model can be of high clinical value. However, a model which strives to be of clinical value requires a thorough sensitivity analysis. In our particular case, the sensitivity analysis needs to determine how the uncertainty in the model parameters affects the primary variables of VGM and DCM, namely the pressure and the mole fraction of dissolved therapeutic agent. In other words, the sensitivity analysis needs to identify the most important influences on the coupled discrete/continuum model for describing cancer therapeutic transport in the lung.
